# Three Decades of Prosthodontic Oral Rehabilitation: A Bibliometric Analysis of Materials, Implants, and Digital Workflows

**DOI:** 10.1155/ijod/7409615

**Published:** 2026-06-19

**Authors:** Yuh-Shan Ho, Abhishek Kumar, Nikolaos Christidis, Anastasios Grigoriadis

**Affiliations:** ^1^ CT HO Trend, 3F.-7, No. 1, Fuxing N. Road, Songshan, Taipei City, 105611, Taiwan; ^2^ Division of Oral Rehabilitation, Department of Dental Medicine, Karolinska Institutet, SE-14104, Huddinge, Sweden, ki.se

**Keywords:** bibliometric analysis, digital technologies, implant therapy, personalized treatment, prosthodontics

## Abstract

**Objective:**

Over the past three decades, prosthodontic research has expanded from material‐ and implant‐focused rehabilitation toward increasingly digital and function‐oriented approaches in oral rehabilitation. Understanding how these shifts align with functional, biological, and patient‐centered rehabilitation goals is essential for future clinical development. To explore these transitions, bibliometric mapping of prosthodontic research published between 1991 and 2024 was performed.

**Methods:**

Data were retrieved from the Web of Science Core Collection (WoSCC) (Science Citation Index Expanded [SCI‐EXPANDED]) using an expanded set of prosthodontics‐related search terms. A “front page” filter (titles, abstracts, and author keywords) was applied to improve specificity. Word frequency analyses were conducted for titles, abstracts, author keywords, and *Keywords Plus*. Structured word cluster analysis was used to identify major research topics, overarching research focuses, and emerging themes. Temporal trends were evaluated across three publication intervals: 1991–2011, 2012−2018, and 2019–2024.

**Results:**

A total of 38,382 articles met the inclusion criteria. Nine main research topics were identified, dominated by implant dentistry, dental materials, and digital dentistry. Four overarching research focuses were delineated: implant‐based rehabilitation, advanced prosthodontic materials, digital and computer‐assisted prosthodontics, and functional rehabilitation. Three emerging themes, namely, 3D printing, accuracy‐driven digital validation, and artificial intelligence (AI), showed marked growth during 2019–2024, reflecting rapid technological adoption.

**Conclusion:**

Prosthodontic research remains strongly anchored in implant therapy and materials science, while digital and data‐driven technologies are expanding rapidly. These trends indicate a transition toward more personalized, efficient, and function‐oriented oral rehabilitation, with implications for future research priorities and clinical practice.

## 1. Introduction

Prosthodontics is a core and one of the oldest dental specialties that integrates science, technology, and clinical care to restore oral function, esthetics, and quality of life in patients with missing or compromised dentition [1]. Central to prosthodontic oral rehabilitation is not only prosthetic replacement but the restoration of effective mastication, occlusal stability, and sensorimotor integration [2–6]. Historically, the field focused on complete and removable dentures, with research emphasizing retention, stability, occlusion, and patient comfort [7]. Over time, advances in materials science, biomechanics, and clinical techniques expanded the scope of prosthodontics, leading to a diversification of research priorities and a gradual shift toward biologically and functionally driven rehabilitation strategies [2].

A key development in this evolution was the introduction of implant dentistry. The introduction of dental implants revolutionized prosthodontics, and the concept of osseointegration provided a reliable and groundbreaking foundation for fixed prosthodontic treatments [8–10]. Implant‐supported restorations consistently demonstrate superior stability and patient satisfaction, establishing implants as a central domain of prosthodontic research and practice [11, 12]. Concurrently, advances in dental materials, including high‐strength ceramics, zirconia, polymer composites, and hybrid systems, have improved the durability, esthetics, and biocompatibility of both tooth‐ and implant‐supported prostheses [13]. However, recent evidence suggests that although dental implants are a clear improvement over conventional removable dentures, they may not fully match the biological and functional performance of natural teeth [2–6].

More recently, technological advances and the rapid integration of digital dentistry and computer‐aided design and manufacturing (CAD/CAM) have represented another defining trend [14]. Digital impressions, cone beam computed tomography (CBCT), and virtual treatment planning facilitate prosthetically driven rehabilitation with high precision [15]. The combination of DICOM data with STL files from intraoral and laboratory scanners enables guided implant surgery and improved prosthetic accuracy while also creating new opportunities for research in occlusion, prosthetic fit, and three‐dimensional biomechanical analysis [16].

In parallel, research in esthetic dentistry, bone regeneration, and tissue engineering supports long‐term implant success and highlights the integration of biological, functional, and technological strategies [17]. Collectively, these developments reflect a clear trend in prosthodontics toward an interdisciplinary, function‐oriented, and digitally integrated approach. However, despite these major scientific and clinical transitions, there is still a limited overview of how prosthodontic research priorities have evolved over time and whether current research adequately reflects the growing emphasis on function, biology, and long‐term rehabilitation outcomes. A bibliometric analysis can help address this gap by systematically mapping publication trends, dominant research themes, and emerging areas of interest, thereby providing a structured basis for future investigations in the field.

Bibliometric analysis has become an established approach for examining research trends, scholarly influence, and the evolution of scientific fields [18, 19]. The Web of Science Core Collection (WoSCC) is widely used for such analyses due to its standardized indexing and comprehensive citation data [20]. In bibliometric studies, the Topic (TS) field typically includes titles, abstracts, and keywords, including both author keywords and *Keywords Plus*, which provide complementary perspectives on research content [20, 21].

Previous methodological developments have further refined bibliometric approaches, including structured mapping, performance analysis, and science mapping techniques [18, 19, 22]. In addition, the “front page” filtering strategy, restricting analyses to titles, abstracts, and author keywords, has been demonstrated to improve specificity and reduce the inclusion of less relevant publications [23].

Against this methodological background and considering the rapid scientific and technological developments in prosthodontics, there is a clear need to systematically map how research priorities have evolved over time. The present study therefore aimed to analyze prosthodontics research indexed in the Science Citation Index Expanded (SCI‐EXPANDED) database from 1991 to 2024 in order to identify major research themes, evaluate temporal changes in research priorities, and detect emerging trends with potential relevance for clinical prosthodontic practice and oral rehabilitation.

## 2. Materials and Methods

### 2.1. Data Collection and Preprocessing

The data for this study were obtained from the WoSCC, the online version of the SCI‐EXPANDED, a commonly used database in bibliometric research [18], with updates as of 21 November 2025. According to the definition of the journal’s impact factor, Chiu and Ho [24] recommended searching documents published in 2024 from SCI‐EXPANDED after the 2024 journal impact factors (IF_2024_) were presented by the Journal Citation Reports (JCR) on 23 June 2025 [24]. The database indexes 9440 journals with citation references across 178 Web of Science categories in SCI‐EXPANDED.

To ensure thorough search coverage, a comprehensive search strategy was developed using quotation marks and Boolean operators (“OR” and “AND”) to capture prosthodontics‐related terminology within the Topic (TS) field. The inclusion criteria were defined as follows: (1) document type limited to original articles, (2) publication years between 1991 and 2024, and (3) presence of relevant prosthodontics‐related terms within the “front page” (title, abstract, or author keywords). Publications not meeting these criteria were excluded from the analysis.

To improve search accuracy and coverage, the keyword strategy was expanded beyond standard prosthodontics terminology to include relevant variations, uncommon expressions, misspelled terms, and terms lacking spaces. All search keywords used in the analysis are listed in Supporting Information 1: File 1.

The full records were exported from the SCI‐EXPANDED database into Microsoft Excel 365 for data management and preprocessing. Data cleaning procedures were performed manually to remove duplicate and irrelevant records prior to analysis in accordance with established bibliometric practices [25]. Word frequency analyses were conducted using structured term extraction from titles, abstracts, author keywords, and *Keywords Plus*. A unified word bank was constructed by integrating terms across these bibliographic fields. Subsequently, word cluster analysis was applied to identify major research topics, overarching research focuses, and emerging trends based on term frequency and co‐occurrence patterns. The WoSCC is primarily designed for literature retrieval rather than direct bibliometric analysis; therefore, appropriate data preprocessing and cleaning procedures are required prior to the analysis to ensure dataset accuracy and relevance.

Preprocessing steps ensured the exclusion of irrelevant records and duplicate entries. Direct export of raw data to bibliometric software was avoided without prior cleaning, consistent with best practices for bibliometric studies. After applying the “front page” filter, 38,382 articles (85% of 44,989 articles) remained, including articles that contain the special terms “post/core restorations,” while 6607 articles (15%) were excluded for lacking the search terms in their “front page” fields. In order to ensure the transparency and reproducibility of the study, the cleaned dataset that was used for the analysis is provided in Supporting Information 2: File 3.

A citation indicator, TC_2024_, the total number of citations received from the WoSCC from the year of publication up to the end of 2024, was applied [26].

### 2.2. Keyword Analysis

Over the past decade and a half, Ho’s and other research groups[20, 26] have conducted extensive investigations into the lexical patterns appearing in article titles, abstracts, author‐supplied keywords, and *Keywords Plus* to identify the main research topics and their trends across diverse scientific domains. These term‐based assessments consistently omit high‐frequency functional words, such as articles, prepositions, and conjunctions, because these terms offer little analytical value for mapping scholarly developments [27]. Using established term‐analysis procedures, the 20 most frequently occurring author keywords, excluding the initial search terms, were identified across four bibliographic components: titles, abstracts, author keywords, and *Keywords Plus*.

### 2.3. Title Word Analysis

In this study, 38,382 prosthodontics‐related articles published between 1991 and 2024 were examined to assess the frequency and distribution of individual words appearing in article titles. The analysis focused exclusively on single‐word terms, a common approach for detecting broad thematic patterns, although it may overlook more nuanced concepts that require multiword expressions. While titles provide a concise indication of a publication’s primary focus, they inherently lack the contextual depth obtainable through full‐text analysis [21].

### 2.4. *Keywords Plus* Analysis

Unlike author keywords, which are intentionally chosen by authors to represent an article’s central themes, *Keywords Plus* are generated algorithmically from recurrent terms found in the titles of cited references [21]. This approach enables the retrieval of a broader range of relevant publications, even when the corresponding terms do not appear in the article’s title, abstract, or author keywords. Nevertheless, a key limitation is that *Keywords Plus* may not accurately reflect the specific content or research focus of the citing article [23]. In some cases, these algorithm‐derived terms may be only loosely connected or entirely unrelated to the main subject matter. Despite this constraint, *Keywords Plus* can still serve as a supplementary vocabulary source, offering insights into broader contextual or background topics within the research field.

### 2.5. Word Cluster Analysis

Word cluster analysis integrates terms extracted from titles, abstracts, author keywords, and *Keywords Plus* into a unified word bank, thereby overcoming the limitations associated with analyzing these bibliographic elements individually [18, 22]. Title and abstract analyses typically rely on single‐word extraction, which may oversimplify complex concepts, and abstract‐derived high‐frequency terms are often broad and lack thematic specificity. Moreover, some articles indexed in the SCI‐EXPANDED database do not include abstracts, author keywords, or *Keywords Plus*, resulting in incomplete or inconsistent data when these elements are examined separately. Additionally, although *Keywords Plus* can capture broader contextual or indirectly related terms, they may not accurately represent the actual content of the citing article. By synthesizing terms across all four sources, word cluster analysis provides a more comprehensive and robust foundation for identifying research themes and mapping intellectual structures within the field.

### 2.6. Topic Identification and Trend Classification Procedure

Following the construction of the unified word bank, a structured procedure was applied to identify the main research topics, research focuses, and emerging trends. First, supporting terms were extracted for each potential topic based on their frequency and co‐occurrence patterns across titles, abstracts, author keywords, and *Keywords Plus*. Articles were then classified as topic‐related if at least one supporting term appeared on the “front page,” ensuring that topic assignment reflected the primary content of each publication. For each topic, the total number of related articles was calculated annually, and temporal patterns were examined across three predefined intervals (1991–2011, 2012−2018, and 2019–2024). Research focuses were identified by grouping closely related topics that demonstrated thematic coherence, while emerging topics were defined as terms or clusters showing marked increases during the most recent interval. This procedure allowed for a systematic evaluation of both long‐standing and rapidly evolving themes in prosthodontic research.

## 3. Results

### 3.1. Shifts in Research Emphasis Relevant to Oral Rehabilitation

A total of 45,310 articles were retrieved in the SCI‐EXPANDED, including 44,989 articles (99% of the 45,310 articles) published between 1991 and 2024 and 321 documents published in 2025. After excluding records not meeting the inclusion criteria and applying the “front page” filter, 38,382 articles were included in the final analysis. To examine changes in research emphasis relevant to prosthodontic oral rehabilitation, 38,382 prosthodontics‐related articles indexed in the SCI‐EXPANDED database between 1991 and 2024 were analyzed. For temporal comparison, the dataset was divided into three approximately equivalent intervals: 1991–2011 (12,482 articles), 2012–2018 (12,323 articles), and 2019–2024 (13,577 articles). The distribution of the 20 most frequently occurring author keywords across these intervals is presented in the accompanying tables, illustrating shifts in research emphasis and the emergence of new directions with potential relevance for clinical practice and oral rehabilitation.

### 3.2. Evolution of Clinically and Functionally Relevant Terminology

Table 1 presents the 20 most frequently occurring title words, excluding the original search terms. Among these, bone was the most prevalent, appearing in 5084 titles (13% of all articles), followed by clinical, found in 3973 titles (10%). Both terms consistently ranked first and second across all three‐time intervals, reflecting their enduring relevance within prosthodontics research.

**Table 1:** Top 20 most frequently used words in the article title during 1991–2024.

Words in title	TP	91–24 *R* (%) *n* = 38,382	91–11 *R* (%) *n* = 12,482	12–18 *R* (%) *n* = 12,323	19–24 *R* (%) *n* = 13,577
Bone	5084	1 (13)	1 (14)	1 (15)	1 (11)
Clinical	3973	2 (10)	2 (11)	2 (10)	2 (10)
Evaluation	2460	3 (6.4)	4 (6.2)	3 (6.8)	5 (6.3)
Surface	2267	4 (5.9)	7 (4.4)	4 (5.9)	3 (7.3)
Patients	1943	5 (5.1)	5 (4.9)	5 (5.3)	8 (5.0)
Report	1874	6 (4.9)	3 (6.9)	7 (4.4)	16 (3.5)
In vitro	1764	7 (4.6)	16 (2.9)	11 (4.1)	4 (6.7)
Mandibular	1541	8 (4.0)	6 (4.5)	9 (4.3)	19 (3.4)
Influence	1512	9 (3.9)	10 (3.6)	10 (4.1)	12 (4.1)
Case	1488	10 (3.9)	8 (4.0)	13 (3.6)	13 (4.0)
Zirconia	1481	11 (3.9)	59 (1.4)	6 (4.4)	6 (5.6)
Maxillary	1476	12 (3.8)	9 (3.8)	8 (4.4)	17 (3.4)
Retrospective	1409	13 (3.7)	29 (2.1)	12 (3.7)	7 (5.0)
Trial	1265	14 (3.3)	53 (1.5)	15 (3.5)	9 (4.8)
Peri‐implant	1247	15 (3.2)	34 (2.0)	16 (3.5)	10 (4.2)
Prospective	1241	16 (3.2)	16 (2.9)	14 (3.6)	22 (3.3)
Immediate	1207	17 (3.1)	12 (3.4)	18 (3.4)	29 (2.7)
Placement	1198	18 (3.1)	15 (3.1)	21 (3.0)	20 (3.3)
Osseointegration	1175	19 (3.1)	31 (2.1)	17 (3.4)	14 (3.6)
Properties	1058	20 (2.8)	49 (1.6)	30 (2.3)	11 (4.2)

*Note*: %, percentage in a period; *R*, rank in a period; TP, total number of articles containing the words on the article title.

Several terms exhibited declining usage over time. For example, “immediate” decreased from 424 occurrences (3.4%, ranked 12th) in 1991–2011 to 368 occurrences (2.7%, ranked 29th) in 2019–2024. Similar downward trends were observed for mandibular, maxillary, and report. Conversely, the term properties demonstrated a substantial rise, occurring 1058 times overall. Its frequency increased from 200 occurrences (1.6%, ranked 49th) in 1991–2011 to 569 occurrences (4.2%, ranked 11th) in 2019–2024. Additional terms showing notable growth in recent years include “vitro,” “trial,” and “peri‐implant,” signaling evolving research interests and the emergence of new focus areas within the field of prosthodontics.

### 3.3. Trends in Research Themes Reflected in Abstracts

Of the 38,382 prosthodontics‐related articles indexed in the SCI‐EXPANDED database from 1991 to 2014, 38,013 (99%) contained abstract information. Word frequency analysis was performed exclusively on these abstracts. Although many of the most frequently occurring terms were broad and lacked the specificity necessary to delineate distinct research themes, their distribution nonetheless provides a useful foundation for identifying general patterns in the field. Despite these limitations, abstract‐level term analysis remains valuable for constructing a baseline vocabulary that can support more targeted evaluations of research trends.

Table 2 reports the 20 most common words found in abstracts from 1991 to 2024, excluding the original search terms. The word bone was the most frequent, appearing in 15,023 abstracts (40% of the 38,013 articles) and maintaining the top rank across all three subperiods (1991–2011, 2012−2018, and 2019–2024). Other terms, such as groups and tests, showed notable increases in recent years, suggesting a heightened emphasis on experimental design, comparative analyses, and assessment methodologies within prosthodontics research.

**Table 2:** Top 20 most frequently used words in the article abstract during 1991–2024.

Words in abstract	TP	91–24 *R* (%) *n* = 38,013	91–11 *R* (%) *n* = 12,211	12–18 *R* (%) *n* = 12,274	19–24 *R* (%) *n* = 13,528
Bone	15,023	1 (40)	1 (40)	1 (42)	1 (37)
Patients	12,944	2 (34)	2 (34)	2 (35)	3 (33)
Significant	11,776	3 (31)	6 (26)	4 (32)	2 (35)
Materials	11,771	4 (31)	5 (27)	3 (34)	5 (31)
Clinical	11,695	5 (31)	3 (29)	5 (30)	4 (33)
Purpose	10,089	6 (27)	4 (28)	6 (29)	12 (24)
Significantly	9291	7 (24)	9 (20)	7 (25)	6 (28)
Surface	9213	8 (24)	7 (21)	8 (25)	8 (27)
Groups	8712	9 (23)	16 (17)	9 (24)	7 (27)
Group	8615	10 (23)	13 (18)	11 (24)	9 (26)
Evaluate	8350	11 (22)	12 (18)	10 (24)	10 (24)
Conclusions	8134	12 (21)	14 (18)	13 (23)	11 (24)
Material	7807	13 (21)	8 (20)	14 (21)	13 (21)
Aim	7516	14 (20)	10 (18)	12 (23)	15 (18)
Test	6676	15 (18)	21 (14)	15 (19)	14 (19)
Patient	6421	16 (17)	15 (17)	16 (18)	17 (16)
Time	6381	17 (17)	18 (17)	17 (17)	16 (17)
Tissue	5997	18 (16)	20 (16)	18 (16)	18 (16)
Months	5952	19 (16)	11 (18)	20 (15)	27 (14)
Teeth	5903	20 (16)	17 (17)	22 (15)	22 (15)

*Note*: %, percentage in a period; *R*, rank in a period; TP, total number of articles containing the words on the article title.

“Bone” was the most frequently used in the titles and abstracts, respectively. The most frequently cited article related to bone contains “bone” in both the title and abstract. The article was “Bone healing and soft tissue contour changes following single‐tooth extraction: A clinical and radiographic 12‐month prospective study” by Schropp et al. [28], which accumulated a TC_2024_ of 1408 citations (ranked 2nd) [28]. This study documented morphological changes in the alveolar bone during the first year after tooth extraction, emphasizing the clinical importance of preserving bone volume to support successful dental implant placement and achieve favorable esthetic and functional outcomes. The authors demonstrated that substantial bone alterations occur within the first postoperative year, underscoring the critical period for managing postextraction bone remodeling.

### 3.4. Author‐Defined Research Priorities in Prosthodontic Rehabilitation

Among the 38,382 prosthodontics‐related articles indexed in the SCI‐EXPANDED, 30,551 (80%) included author‐defined keywords. Consequently, keyword analysis in this study was performed on this subset. Although this represents a smaller proportion of the full dataset, author keywords provide notable advantages. Because they are intentionally selected by the authors, these terms tend to be concise, topic‐specific, and directly aligned with the central themes of each publication. In contrast, abstract‐based word analysis, conducted on 38,013 articles, offers broader textual coverage but often includes generic or nonspecific vocabulary, making it less effective for identifying well‐defined research topics. Thus, author keywords serve as a more focused and reliable indicator of emerging trends and thematic developments within prosthodontics.

Table 3 summarizes the 20 most frequently used author keywords, excluding original search terms, in prosthodontics‐related articles published from 1991 to 2024. The most frequently used author keyword was “osseointegration,” which appeared in 2136 articles (7.0% of those with author keyword data). The most highly cited article employing this keyword was “Long‐term evaluation of nonsubmerged ITI implants. Part 1: 8‐year life table analysis of a prospective multicenter study with 2359 implants” by Buser et al. [29], which accumulated a TC_2024_ of 1033 citations (ranked 9th) [29]. This multicenter investigation followed 2359 nonsubmerged ITI implants for up to 8 years and reported excellent long‐term outcomes, with survival and success rates of 96.7% and 93.3%, respectively. Early failures were uncommon, and screw‐type and mandibular implants demonstrated the most favorable performance. 5‐year results further verified the robustness of the findings, showing consistently high success across participating centers.

**Table 3:** Top 20 most frequently used author keywords during 1992–2024.

Author keywords	TP	91–24 *R* (%) *n* = 30,551	91–11 *R* (%) *n* = 8439	12–18 *R* (%) *n* = 10,261	19–24 *R* (%) *n* = 11,851
Osseointegration	2,136	1 (7.0)	1 (7.9)	1 (7.0)	1 (6.3)
Peri‐implantitis	1,016	2 (3.3)	9 (1.6)	3 (3.0)	2 (4.9)
Zirconia	953	3 (3.1)	7 (1.8)	2 (3.5)	3 (3.8)
Finite element analysis	767	4 (2.5)	4 (2.3)	5 (2.4)	4 (2.8)
Immediate loading	610	5 (2.0)	2 (2.7)	4 (2.4)	19 (1.1)
Bone regeneration	593	6 (1.9)	5 (1.9)	6 (1.9)	6 (2.0)
Hydroxyapatite	467	7 (1.5)	3 (2.5)	13 (1.1)	18 (1.2)
Bone	395	8 (1.3)	6 (1.9)	7 (1.6)	61 (0.58)
CAD/CAM	373	9 (1.2)	26 (0.81)	8 (1.5)	16 (1.2)
Surface modification	372	10 (1.2)	13 (1.1)	10 (1.2)	14 (1.3)
Biomechanics	344	11 (1.1)	8 (1.7)	12 (1.2)	45 (0.71)
Biomaterials	340	12 (1.1)	18 (0.90)	9 (1.3)	21 (1.1)
Biocompatibility	337	13 (1.1)	14 (1.1)	28 (0.87)	13 (1.3)
Dental prosthesis	326	14 (1.1)	42 (0.66)	15 (1.1)	14 (1.3)
Dental materials	314	15 (1.0)	19 (0.89)	51 (0.64)	9 (1.5)
Mandible	309	16 (1.0)	11 (1.2)	11 (1.2)	46 (0.69)
Histology	307	17 (1.0)	10 (1.5)	13 (1.1)	77 (0.53)
Mechanical properties	300	18 (1.0)	34 (0.72)	39 (0.76)	11 (1.4)
Accuracy	295	19 (1.0)	233 (0.21)	62 (0.58)	7 (1.8)
3D printing	289	20 (0.95)	N/A	186 (0.28)	5 (2.2)

*Note*: %, percentage in a period; *R*, rank in a period; TP, total number of articles containing the words on the article title.

Abbreviation: N/A, not available.

Several emerging keywords exhibited substantial growth across the study period. “3D printing” appeared in 289 articles overall and rose sharply from no occurrences in 1991–2011 to 29 articles (0.28%, ranked 186th) in 2012–2018 and 260 articles (2.2%, ranked 5th) in 2019–2024, demonstrating rapidly increasing scholarly attention to additive manufacturing technologies in prosthodontics. Similar upward trajectories were observed for “accuracy,” “dental prosthesis,” and “mechanical properties,” reflecting the expanding research interest in these areas. Conversely, the keyword “histology” showed a pronounced decline, decreasing from 126 articles (1.5%, ranked 10th) in 1991–2011 to 63 articles (0.53%, ranked 77th) in 2019–2024. Furthermore, “artificial intelligence” and “deep learning” can only be used as author keywords in 2019–2024.

### 3.5. Contextual Research Themes Identified Through *Keywords Plus*

Among the 38,382 prosthodontics‐related articles indexed in the SCI‐EXPANDED database, 35,039 (91%) contained *Keywords Plus*. Table 4 summarizes the 20 most frequently occurring *Keywords Plus* terms, excluding original search terms, in prosthodontics‐related literature published between 1991 and 2024. The most common terms were “bone” (2888 articles, 8.2%), “in vitro” (2227 articles, 6.4%), “placement” (1715 articles, 4.9%), and “osseointegration” (1570 articles, 4.5%).

**Table 4:** Top 20 most frequently used *Keywords Plus* during 1991–2024.

*Keywords Plus*	TP	91–24 *R* (%) *n* = 35,039	91–11 *R* (%) *n* = 10,693	12–18 *R* (%) *n* = 11,753	19–24 *R* (%) *n* = 12,593
Bone	2888	1 (8.2)	1 (10)	2 (8.4)	1 (7.0)
In vitro	2227	2 (6.4)	2 (5.2)	1 (9.1)	3 (4.8)
Placement	1,715	3 (4.9)	4 (5.0)	5 (5.0)	4 (4.7)
Osseointegration	1570	4 (4.5)	11 (2.8)	3 (5.5)	2 (5.0)
Follow‐up	1374	5 (3.9)	5 (3.7)	4 (5.4)	15 (2.8)
Surface	1226	6 (3.5)	12 (2.7)	6 (3.5)	7 (4.2)
Strength	1168	7 (3.3)	8 (3.2)	8 (3.1)	9 (3.6)
Teeth	1112	8 (3.2)	6 (3.6)	7 (3.3)	18 (2.7)
Accuracy	1086	9 (3.1)	31 (1.7)	11 (2.8)	5 (4.6)
Mechanical properties	1008	10 (2.9)	37 (1.5)	17 (2.5)	6 (4.4)
Behavior	997	11 (2.8)	24 (1.9)	13 (2.7)	8 (3.8)
Stability	948	12 (2.7)	27 (1.8)	9 (3.0)	10 (3.3)
Surgery	905	13 (2.6)	14 (2.5)	15 (2.6)	20 (2.6)
Osseointegrated implants	900	14 (2.6)	3 (5.2)	28 (2.1)	136 (0.75)
Reconstruction	854	15 (2.4)	9 (3.2)	22 (2.3)	35 (1.9)
Differentiation	851	16 (2.4)	32 (1.6)	10 (2.9)	17 (2.7)
Adhesion	848	17 (2.4)	26 (1.8)	11 (2.8)	19 (2.6)
Complications	848	17 (2.4)	29 (1.7)	14 (2.7)	12 (2.8)
Augmentation	823	19 (2.3)	22 (2.2)	16 (2.6)	26 (2.3)
Interface	790	20 (2.3)	7 (3.5)	25 (2.2)	66 (1.3)

*Note*: %, percentage in a period; *R*, rank in a period; TP, total number of articles containing the words on the *Keywords Plus*.

Several keywords demonstrated increasing prominence over time, including “mechanical properties” (1008 articles) and “accuracy” (1086 articles), reflecting growing research attention to material performance and measurement precision. Conversely, other terms, such as “osseointegrated implants” (ranked 14th, 2.6%), “interface” (20th, 2.3%), and “reconstruction” (15th, 2.4%), exhibited declining use over the study period.

Overall, while *Keywords Plus* must be interpreted cautiously due to their indirect connection to article content, their analysis provides complementary insights into prevailing themes and shifting research directions within the prosthodontics literature.

### 3.6. Major Research Domains Shaping Prosthodontic Oral Rehabilitation

**Figure 1:**
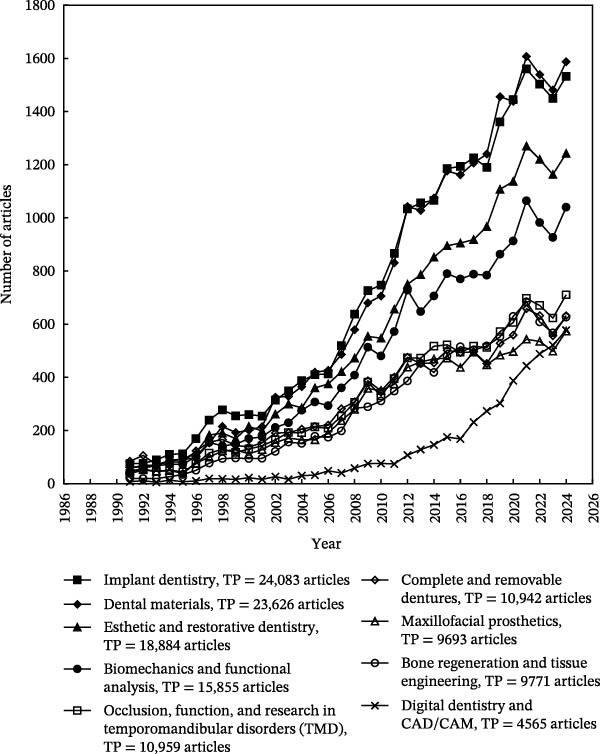
The development trends of nine research topics on prosthodontic research.

**Figure 2:**
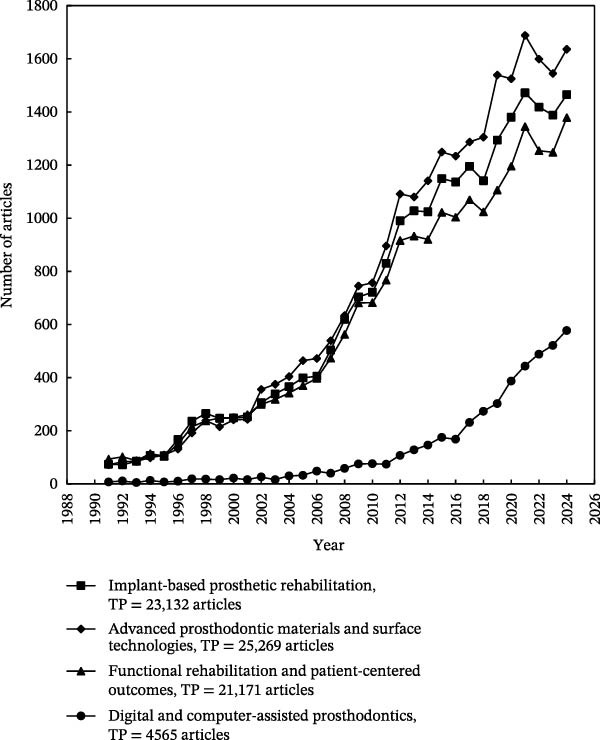
The development trends of the four main research foci on prosthodontic research.

**Figure 3:**
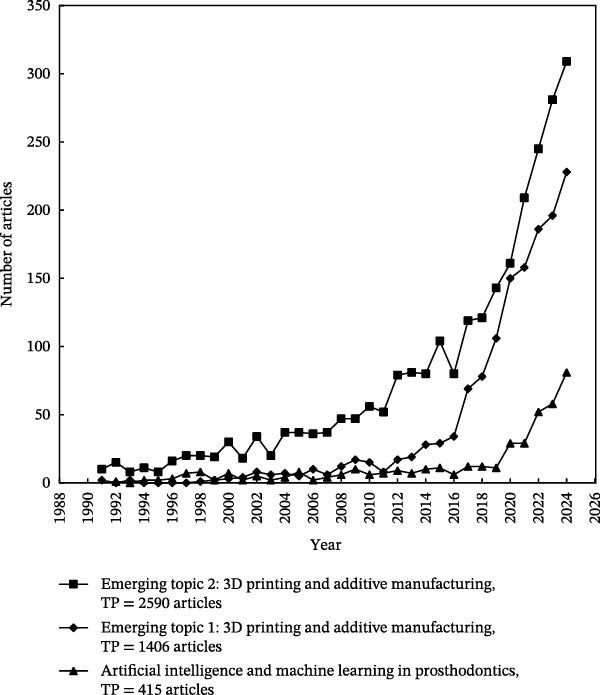
The development trends of three emerging research foci in prosthodontics research.

The word cluster analysis was carried out through a structured, multistep approach designed to systematically identify and contextualize major research themes within the prosthodontics literature:

1. Identification of core subjects and topics:
 Using their professional expertise, three domain experts (Anastasios Grigoriadis, Abhishek Kumar, and Nikolaos Christidis) collaboratively evaluated the word frequency analysis results as a word bank, capturing broader linguistic patterns to identify both primary and emerging research focuses within the field. Topic identification and classification were based on consensus to minimize individual bias.
2. Extraction of supporting terms:
 Supporting words, which help define primary and emerging research topics, were identified from the comprehensive word bank compiled by integrating terms across multiple bibliographic sources, including article titles, abstracts, author keywords, and *Keywords Plus*.
3. Classification of topic‐related articles:
 Articles were classified as topic‐specific if at least one supporting term from the corresponding topic cluster appeared on the article’s “front page,” that is, within the title, abstract, or author keywords. This filtering ensured that only publications with clear relevance to the identified themes were included in subsequent analyses, enhancing the reliability of topic assignments. The number of topic‐related articles was calculated annually, and these data were used to generate trend figures illustrating the temporal development of each research topic (Figures 1–3).

The research topics in prosthodontic research (Figure 1):

• Topic 1: implant dentistry:
 Implant dentistry encompasses research on the biological, mechanical, and clinical foundations of implant‐supported rehabilitation. This topic includes studies on osseointegration, peri‐implant tissue behavior, implant placement protocols, and long‐term success rates. The clustering is strongly supported by the dominance of implant‐related terms across titles, abstracts, author keywords, and *Keywords Plus*, indicating that implants remain the central axis of prosthodontic research.
 Supporting words: bone, osseointegration, peri‐implantitis, peri‐implant, placement, stability, follow‐up, behavior, augmentation, and complications.
• Topic 2: dental materials:
 This topic integrates research on the development, characterization, and clinical performance of dental materials used in prosthodontics. It includes investigations of ceramics, polymers, composites, and surface treatments, often linking laboratory outcomes to clinical applicability. The grouping is justified by the consistently high frequency of material‐related terminology in both abstracts and author keywords, reflecting a major research pillar.
 Supporting words: materials, zirconia, mechanical properties, hydroxyapatite, biocompatibility, biomaterials, surface modification, strength, adhesion, vitro.
• Topic 3: digital dentistry and CAD/CAM:
 Digital dentistry includes studies on CAD/CAM, intraoral scanning, digital workflows, and precision‐driven restorative fabrication. The rising prevalence of digital terms, particularly in the most recent period, supports grouping these concepts as a distinct and rapidly expanding topic. This cluster captures the technological shift that is transforming prosthodontic practice.
 Supporting words: CAD/CAM, accuracy, 3D printing, digital workflow, intraoral scanning, milling, additive manufacturing, virtual design, scanning accuracy, and digital impression.
• Topic 4: biomechanics and functional analysis:
 This topic comprises research exploring the mechanical behavior of prosthetic components, occlusal loading, stress distribution, and structural optimization. It is frequently grounded in computational simulations, especially in finite element analysis. The thematic grouping derives from the repeated co‐occurrence of biomechanics‐related terms that emphasize functional and mechanical considerations rather than biological ones.
 Supporting words: finite element analysis, biomechanics, influence, behavior, stress, stability, loading, mandible mechanics, interface, and deformation.
• Topic 5: maxillofacial prosthetics:
 Maxillofacial prosthetics focuses on rehabilitating patients with congenital or acquired craniofacial defects through extraoral and intraoral prostheses. The topic incorporates reconstructive principles, material choices, and clinical outcomes specific to maxillofacial rehabilitation. High‐frequency anatomical terms and reconstruction‐related terminologies consistently cluster these studies together.
 Supporting words: maxillary, mandibular, reconstruction, maxillofacial prosthetics, facial prosthesis, defect rehabilitation, orbital prosthesis, tissue replacement, esthetic restoration, and craniofacial reconstruction.
• Topic 6: complete and removable dentures:
 This topic includes research on the design, fabrication, biomechanics, and patient outcomes of complete and partial removable dentures. Studies often emphasize clinical trials, functional evaluation, and material performance in traditional prosthetic treatments. The grouping is supported by recurrent patient‐centered terms associated with denture studies in abstracts and clinical titles.
 Supporting words: denture, removable prosthesis, complete denture, overdenture, clinical evaluation, retention, stability, tissue adaptation, occlusal scheme, and patient satisfaction.
• Topic 7: occlusion, function, and research in temporomandibular disorders (TMDs):
 This topic captures investigations into occlusal relationships, temporomandibular joint (TMJ) function, mandibular dynamics, and their relationship to prosthetic rehabilitation. The clustering is justified by the recurring presence of anatomical and functional terms, especially “mandibular,” “maxillary,” and terms related to functional evaluation, across multiple bibliographic fields.
 Supporting words: occlusion, mandibular movement, TMD, TMJ, function, maxillary, bite force, group function, articulation, TMDs, and jaw biomechanics.
• Topic 8: esthetic and restorative dentistry:
 This topic encompasses research on restorative procedures emphasizing esthetics, including ceramic restorations, veneer systems, and color‐matching technologies. It includes laboratory and clinical studies evaluating the surface properties, translucency, and clinical success of esthetic materials. The grouping is driven by zirconia‐ and surface‐related frequencies, which consistently co‐occur in restorative contexts.
 Supporting words: zirconia, ceramic restoration, esthetics, surface, translucency, veneer, shade matching, polishing, evaluation, and surface modification.
• Topic 9: bone regeneration and tissue engineering:
 Research in this topic covers bone augmentation procedures, grafting materials, cellular responses, and biologically driven regeneration strategies. Clustering is justified by the strong presence of regeneration‐, differentiation‐, and augmentation‐related terminology that frequently appears together with implant‐related terms but also forms a distinct subfield with its own focus.
 Supporting words: bone regeneration, augmentation, differentiation, adhesion, tissue engineering, graft materials, osteogenesis, scaffold, healing, and growth factors.

The main research foci on prosthodontic research (Figure 2):

• Research focus 1: implant‐based prosthetic rehabilitation:
 Implant‐based prosthetic rehabilitation represents the central research focus within prosthodontics, integrating the biological, clinical, and mechanical dimensions of implant therapy. Studies within this focus examine osseointegration, peri‐implant tissue behavior, surgical protocols, long‐term survival, and complication management. The grouping is strongly supported by the dominance of implant‐related terminology, in particular, “bone,” the most frequent term across all bibliographic fields, demonstrating the enduring prominence of implant dentistry in the discipline.
 Supporting words: bone, osseointegration, peri‐implantitis, peri‐implant, placement, stability, follow‐up, complications, augmentation, and bone regeneration.
• Research focus 2: advanced prosthodontic materials and surface technologies:
 This focus area encompasses the development, characterization, and clinical assessment of contemporary restorative materials and surface technologies. Research topics within this cluster include zirconia, ceramics, biocompatibility, surface modification, and mechanical performance. The grouping is justified by the consistently high frequency of material‐related terms, such as “materials,” “zirconia,” and “mechanical properties,” which collectively underscore the field’s strong emphasis on improving restorative durability, esthetics, and biological integration.
 Supporting words: materials, zirconia, mechanical properties, surface, surface modification, strength, hydroxyapatite, biocompatibility, adhesion, and biomaterials.
• Research focus 3: digital and computer‐assisted prosthodontics:
 Digital prosthodontics represents a rapidly expanding focus, integrating CAD/CAM technologies, digital workflows, intraoral scanning, and precision‐driven fabrication techniques. Research in this area increasingly focuses on accuracy, reproducibility, digital impression methods, and additive manufacturing. The substantial rise of digital‐related terminology, especially the steep increase in “accuracy” and “3D printing” in the period 2019–2024, supports classifying this field as a major contemporary focus of prosthodontic innovation.
 Supporting words: CAD/CAM, accuracy, 3D printing, digital workflow, intraoral scanning, additive manufacturing, virtual design, milling, digital impression, and scanning precision.
• Research focus 4: functional rehabilitation and patient‐centered outcomes:
 This focus area synthesizes research on occlusal function, TMJ dynamics, denture performance, and patient‐reported outcomes. It reflects the clinical emphasis on restoring function, comfort, and oral health‐related quality of life. The grouping is supported by recurrent high‐frequency terms related to patients, teeth, clinical evaluation, mandibular dynamics, and occlusal concepts, indicating the sustained relevance of functional assessment within prosthodontic care.
 Supporting words: patients, clinical evaluation, occlusion, mandibular, maxillary, TMJ, bite force, complete denture, stability, and patient satisfaction.

The emerging research foci in prosthodontic research (Figure 3):

• Emerging research focus 1: 3D printing and additive manufacturing:
 3D printing has rapidly evolved from an experimental technique to a central innovation in prosthodontic research, enabling customizable, highly precise restorative, and surgical solutions. The exponential increase in author keyword frequency, especially between 2019 and 2024, highlights this domain as the most prominent emerging frontier. Its thematic independence and strong temporal growth justify its classification as a standalone emerging topic.
 Supporting words: 3D printing, additive manufacturing, printing accuracy, digital fabrication, resin materials, build orientation, layer thickness, printed prosthesis, rapid prototyping, and digital workflow.
• Emerging research focus 2: accuracy‐ and precision‐driven digital prosthodontics:
 This emerging topic captures the research surge centered around validating the precision of digital tools, including scanners, milling units, and CAD/CAM systems. Accuracy‐related terminology has shown one of the steepest ranking improvements across author keywords and *Keywords Plus*, reflecting a field‐wide emphasis on reproducibility and measurement reliability. The growth pattern indicates a paradigm shift toward quantitative performance validation in digital dentistry.
 Supporting words: accuracy, precision, trueness, reproducibility, deviation analysis, scanning accuracy, fit assessment, measurement error, marginal adaptation, and digital validation.
• Emerging research focus 3: artificial intelligence (AI) and machine learning (ML) in prosthodontics:
 AI and ML have recently emerged as transformative tools for diagnostic support, automated design, predictive modeling, and outcome assessment. Although the overall frequency remains comparatively low, the restriction of these terms to the most recent period (2019–2024) indicates the birth of a new research frontier. Its emergence aligns with broader technological integration trends observed across biomedical fields.
 Supporting words: AI, deep learning, ML, neural networks, automated design, predictive modeling, image analysis, decision support, algorithm training, and data‐driven prosthodontics.

## 4. Discussion

The synthesis of the identified research topics, major focuses, and emerging trends illustrates a prosthodontic field undergoing rapid scientific, technological, and societal transformation. Implant‐based prosthetic rehabilitation continues to dominate the research landscape, reflecting decades of evidence supporting predictable osseointegration, improved peri‐implant tissue management, and long‐term survival outcomes [28, 29]. Parallel to this, the strong emphasis on advanced materials, in particular zirconia, high‐strength ceramics, and engineered surfaces, aligns with ongoing efforts to enhance mechanical durability, biocompatibility, and esthetic stability, as demonstrated in contemporary prosthodontic materials research [30–36]. The rise of digital and computer‐assisted prosthodontics represents a fundamental reorganization of clinical workflows, driven by economic pressures for efficiency, reduced laboratory time, and the increasing availability of high‐precision digital fabrication technologies. Recent studies show that digital workflows improve accuracy, reduce production costs, and enhance reproducibility [15, 37–39]. However, increased technical accuracy does not necessarily translate into improved oral function, and the functional and sensorimotor consequences of digitally optimized prosthetic workflows remain insufficiently explored. From a clinical oral rehabilitation perspective, this pattern reflects a broader prioritization of structural stability and prosthetic survival over functional integration, sensorimotor adaptation, and patient‐centered outcomes.

At the same time, demographic and societal factors markedly influence research priorities. As populations age while remaining healthier and more functionally active, patient expectations for esthetic, functional, and long‐lasting prosthetic solutions have intensified. Literature on geriatric prosthodontics consistently demonstrates that today’s older adults demand higher oral function, improved esthetics, and treatments that support quality of life well into an advanced age [40, 41]. These expectations place new pressures on clinicians and researchers to develop minimally invasive, cost‐effective, and esthetically optimized therapies. Emerging topics, including 3D printing, accuracy‐driven digital validation, and initial applications of AI, reflect the field’s response to these evolving demands. Additive manufacturing enables individualized prostheses with reduced cost and fabrication time [42–44], while AI‐supported diagnostic and design tools promise improved precision and predictive modeling in prosthodontic rehabilitation [45]. From a rehabilitation standpoint, these technologies hold potential not only for personalized fabrication but also for future integration of functional prediction, adaptive prosthetic design, and patient‐specific rehabilitation strategies. Collectively, these developments demonstrate that prosthodontic research is increasingly shaped by technologies that may enable more individualized and functionally informed oral rehabilitation. The convergence of these factors positions prosthodontics at the forefront of personalized, digitally enabled, and biologically informed oral rehabilitation.

In addition to the identified emerging trends, several clinically relevant technologies may currently be underrepresented in bibliometric analysis. Innovations such as facial scanning systems, jaw tracking devices, motion analysis technologies, and patient‐specific or subperiosteal implant solutions are increasingly integrated into prosthodontic workflows, particularly in function‐oriented and personalized rehabilitation. However, these developments may not yet be fully captured in keyword‐based analyses due to the variability in terminology and relatively low frequency of use in the literature. This highlights an inherent limitation of bibliometric approaches, where rapidly evolving and highly specialized clinical innovations may remain under‐detected despite their growing clinical importance.

### 4.1. Strengths and Limitations

This study has several notable strengths. First, it provides a comprehensive and long‐term bibliometric overview of prosthodontic research spanning over three decades. By analyzing a large dataset of over 38,000 articles indexed in the SCI‐EXPANDED, the study captures both historical foundations and contemporary developments within the field. The use of the “front page” filtering strategy, restricting analyses to terms appearing in the title, abstracts, and author keywords, improves specificity and minimizes the inclusion of publications with only marginal relevance to prosthodontics.

A further strength lies in the multilayered analytical approach. By integrating word frequency analyses across titles, abstracts, author keywords, and *Keywords Plus* and subsequently applying structured word cluster analysis, this study offers a robust mapping of the intellectual structure of prosthodontic research. The identification of nine major research topics, four overarching research focuses, and three emerging research areas provides a coherent framework that reflects both established domains, such as implant dentistry and materials science, and rapidly evolving technological frontiers, including digital workflows, 3D printing, and AI. Dividing the study period into three balanced temporal intervals further enabled a systematic assessment of thematic evolution and reduced bias related to short‐term publication fluctuations.

The methodological transparency of the bibliometric procedures represents an additional strength. Detailed reporting of data collection, preprocessing, keyword selection, and topic classification enhances reproducibility and allows for meaningful comparison with other bibliometric studies using similar approaches. Moreover, the focus on terminology evolution across bibliographic components provides insights not only into research volume but also into conceptual and methodological shifts within prosthodontics. Finally, this bibliometric study was designed and reported in accordance with the BIBLIO checklist (Supporting Information 3: File 2).

Several limitations should nevertheless be acknowledged. First, the analysis was restricted to the WoSCC, which, although comprehensive and widely used, does not include all prosthodontics‐related publications. The use of a single database was a deliberate methodological choice as bibliometric analyses require internally consistent data structures, including citation metrics, *Keywords Plus*, and subject categorization, which differ substantially across databases such as Web of Science, Scopus, and PubMed. Combining multiple databases may, therefore, introduce inconsistencies and reduce comparability of results. However, restricting the analysis to one database may still introduce some degree of geographic or thematic bias, particularly by underrepresenting studies indexed exclusively in other databases or non‐English‐language journals.

In addition, the present study was designed as a content‐based bibliometric analysis focusing on thematic development and terminological patterns. Therefore, performance‐based indicators such as leading authors, institutions, countries, and journal productivity were not included. While these metrics may provide complementary insights, they represent a different analytical dimension and were beyond the scope of this study.

Second, bibliometric analyses inherently rely on publication metadata rather than the full‐text content. Although the use of front‐page elements improves relevance, it may still overlook nuanced or emerging concepts that are not explicitly reflected in the titles, abstracts, or keywords. In addition, citation‐based indicators reflect research visibility and dissemination rather than intrinsic scientific quality or clinical impact and may favor older publications, highly cited review articles, or technologically popular topics.

Finally, the identification and classification of research topics through word clustering involve a degree of expert interpretation. Although the process was conducted collaboratively by three experts and based on consensus and supported by systematic frequency and co‐occurrence patterns, no formal interreviewer agreement statistics or algorithm‐based validation methods were applied. Consequently, some degree of subjectivity and potential classification bias cannot be completely excluded.

## 5. Conclusion

This study provides an overview of the temporal evolution of prosthodontic research over the past three decades, revealing both long‐standing priorities and rapidly emerging research directions. Implant‐based rehabilitation and advanced prosthodontic materials continue to dominate the literature, while digital and computer‐assisted workflows have increasingly shaped research and clinical practice.

From an oral rehabilitation perspective, these trends highlight a growing need to align technological innovation with functional, biological, and patient‐centered outcomes. While digitalization, additive manufacturing, and AI offer substantial potential for precision and personalization, their clinical value will ultimately depend on how effectively they contribute to restored oral function, adaptive sensorimotor integration, and long‐term rehabilitation outcomes. Future prosthodontic research should therefore prioritize integrative approaches that combine technological advances with function‐oriented and patient‐centered rehabilitation strategies.

## Author Contributions

Conceptualization: Yuh‐Shan Ho, Anastasios Grigoriadis, Abhishek Kumar, and Nikolaos Christidis. Methodology: Yuh‐Shan Ho, Anastasios Grigoriadis, Abhishek Kumar, and Nikolaos Christidis. Formal analysis: Yuh‐Shan Ho. Investigation: Yuh‐Shan Ho, Anastasios Grigoriadis, Abhishek Kumar, and Nikolaos Christidis. Data curation: Yuh‐Shan Ho. Writing – original draft preparation: Nikolaos Christidis and Abhishek Kumar. Writing – review and editing: Yuh‐Shan Ho and Anastasios Grigoriadis.

## Funding

This study did not receive any specific funding.

## Disclosure

All authors have read and approved the final version of the manuscript. Nikolaos Christidis and all authors had full access to all of the data in this study and take complete responsibility for the integrity of the data and the accuracy of the data analysis. The research conducted in this manuscript is original, not presently under consideration for publication elsewhere, and free of conflicts of interest. The authors alone are responsible for the content and writing of the paper.

## Ethics Statement

Ethical approval was not required for this study as it was based exclusively on publicly available bibliometric data and did not involve human participants or identifiable personal data.

## Consent

Not applicable since no patient data were reported.

## Conflicts of Interest

The authors declare no conflicts of interest.

## Supporting Information

Additional supporting information can be found online in the Supporting Information section.

## Supporting information


**Supporting Information 1** File 1: Used search keywords for the bibliometric analysis.

**Supporting Information 2** File 3: The cleaned dataset that was used for the analysis. This Supporting Information file contains the cleaned dataset used for the present bibliometric analysis. The dataset, derived from the Web of Science Core Collection (SCI‐EXPANDED), includes the records that met the predefined inclusion criteria. In this dataset the bibliographic information such as publication year, title, journal, author keywords, *Keywords Plus*, and other relevant metadata used for the analyses are included.

**Supporting Information 3** File 2: The BIBLIO checklist for reporting the bibliometric reviews of the biomedical literature.

## Data Availability

Data generated and/or analyzed during the current study are freely available from the Web of Science. The authors confirm that the data supporting the findings of this study are available within the article and/or its Supporting Information, so the cleaned dataset used for the analysis is provided as Supporting Information 2: File 3.
