# Supplementary material for: Three Decades of Prosthodontic Oral Rehabilitation: A Bibliometric Analysis of Materials, Implants, and Digital Workflows
Source: Int J Dent. 2026 Jun 19;2026:7409615. doi: 10.1155/ijod/7409615 (PMC13280661; doi:10.1155/ijod/7409615)
Supplement: Supplementary file 1 — Supporting Information 1 File 1: Used search keywords for the bibliometric analysis. [file IJOD-2026-7409615-s002.docx]

**Supplementary file 1.** Used search keywords

“dental implants,” “dental implant,” “titanium implants,” “partial dentures,” “dental prostheses,” “prosthodontic,” “overdentures,” “titanium implant,” “prosthodontics,” “partial denture,” “dental restorations,” “overdenture,” “ceramic restorations,” “dental restoration,” “oral rehabilitation,” “tooth replacement,” “implant-supported prostheses,” “prosthetic treatment,” “all-ceramic crowns,” “implant-supported prosthesis,” “ceramic implants,” “dental crowns,” “dental crown,” “ceramic restoration,” “all-ceramic crown,” “ceramic implant,” “implant supported crowns,” “tooth replacements,” “implant supported crown,” “maxillofacial prosthetics,” “maxillofacial prosthetic,” “cosmetic dentistry,” “full dentures,” “prosthetic treatments,” “implant-supported dentures,” “dental bridges,” “dental bridge,” “full denture,” “implant-supported denture,” “post-core restorations,” “implant supported bridges,” “implant supported bridge,” “post-core restoration,” and “survival and success crowns”
